# Outcome of initial cord blood transplantation with FM80TBI as conditioning regimen for acute myeloid leukemia

**DOI:** 10.1007/s00277-026-06888-3

**Published:** 2026-02-16

**Authors:** Masahiro Akimoto, Takayoshi Tachibana, Masatsugu Tanaka, Akihiko Izumi, Takaaki Takeda, Katsumichi Fujimaki, Yotaro Tamai, Shuku Sato, Hiroyuki Fujita, Takahiro Suzuki, Koh Yamamoto, Etsuko Yamazaki, Nodoka Maeda, Shota Arai, Natsuki Hirose, Hideaki Nakajima

**Affiliations:** 1 Department of Hematology, Kanagawa Cancer Center, 2-3-2, Nakao, Asahi-ku, Yokohama, 241-8515 Kanagawa Japan; 2 Department of Hematology, Fujisawa City Hospital, Fujisawa, Japan; 3 Division of Hematology, Shonan Kamakura General Hospital, Kamakura, Japan; 4 Department of Hematology, Saiseikai Yokohama Nanbu Hospital, Yokohama, Japan; 5 Department of Hematology, Kitasato University School of Medicine, Sagamihara, Japan; 6 Department of Hematology, Yokohama City Minato Red Cross Hospital, Yokohama, Japan; 7 Department of Hematology, Yokohama Rosai Hospital, Yokohama, Japan; 8 Division of Hematology, Department of Medicine, Showa University Fujigaoka Hospital, Yokohama, Japan; 9 Department of Stem Cell and Immune Regulation, Yokohama City University Graduate School of Medicine, Yokohama, Japan

**Keywords:** Acute myeloid leukemia, Cord blood transplant, Fludarabine, Melphalan, Total-body irradiation

## Abstract

Outcomes of allogeneic stem cell transplantation in older patients with acute myeloid leukemia (AML) have been unsatisfactory. At our center, cord blood transplantation (CBT) using FM80TBI has been implemented in older patients or those with comorbidities. We conducted a retrospective single-center study to evaluate transplantation outcomes in patients with AML who underwent CBT following FM80TBI. The FM80TBI regimen consisted of fludarabine (125 or 150 mg/m²), melphalan (80 mg/m²), and total-body irradiation (4 Gy). This study included 58 patients. The median follow-up period was 38 months. The median age at transplant was 67 (52–73) years. At the time of CBT, 39.7% of all patients having an Eastern Cooperative Oncology Group performance status of ≤ 2 did not achieve complete remission (CR), 25.9% had adverse cytogenetic risk, and 53.4% were positive for Wilms tumor gene-1 messenger RNA in peripheral blood. Neutrophil engraftment by day 42 was achieved in 87.9% of patients. The 3-year overall survival (OS) was 67.1%, the 3-year non-relapse mortality was 14.4%, and the 3-year cumulative incidence of relapse was 20.0%. Patients with CR status at the time of CBT had significantly better 3-year OS than those with non-CR status (86.8% vs. 42.4%, *P* < 0.05). The cumulative incidences of Grade II–IV acute graft-versus-host disease (GVHD) at day 100 and chronic GVHD at 2 years were 29.4% and 15.0%, respectively. Although this was a single-center retrospective study, the outcomes of CBT for older patients with AML or those with comorbidities following FM80TBI were favorable.

**Supplementary Information:**

The online version contains supplementary material available at 10.1007/s00277-026-06888-3.

## Introduction

Acute myeloid leukemia (AML) predominantly affects older adults, with a median age at diagnosis of 68 years [1]. Managing older patients with AML is often challenging owing to age-related non-hematologic comorbidities and their increased vulnerability to therapeutic toxicities. Hematopoietic stem cell transplantation (HCT) remains the only treatment approach with the potential to achieve long-term survival.

Cord blood transplantation (CBT) offers an alternative source of HCT for patients with curable hematologic diseases requiring allogenic HCT [2]. The introduction of CBT has expanded access to allogenic HCT, especially for older patients requiring urgent HCT. Older patients undergoing myeloablative conditioning for HCT may have unacceptable treatment-related mortality [3]. The advent of reduced-intensity conditioning (RIC) has increased access to HCT [4]. RIC regimens typically consist of alkylating agents, such as busulfan or melphalan, combined with fludarabine (FM) [5]. Fludarabine combined with melphalan at a dose of 140 mg/m² (FM140), developed two decades ago, is one of the most widely used RIC regimens [6]. Low-dose total body irradiation (TBI) had been commonly added to FM because TBI enhances neutrophil recovery in CBT [7]. Japanese real-world data analysis revealed an excellent survival outcome for FM140TBI among fludarabine-based conditioning regimens in CBT [8]. However, studies have shown that FM was linked with higher antitumor effects but slightly more severe toxicities than fludarabine and busulfan [9]. This concern has prompted transplant physicians to opt for lower-dose FM, especially for older patients and those with significant comorbidities, to reduce treatment-related toxicity. At our center, CBT with FM80TBI has been adopted for older patients or those with complications. To our knowledge, there are no reports on the outcomes of initial CBT with FM80TBI for AML. This study aimed to evaluate the transplantation outcomes of patients with AML who underwent initial CBT after FM80TBI.

## Materials and methods

### Study design and patients

This was a single-center, retrospective study. The eligibility criteria included patients who underwent their first CBT between January 2015 and June 2024 following 25 or 30 mg/m^2^/day of fludarabine for 5 days and 40 mg/m^2^/day of melphalan for 2 days with 4 Gy of TBI as a conditioning regimen for AML. The preconditioning intervention (PCI) refers to chemotherapy applied to patients with high-risk AML in routine, patient-oriented clinical practice. According to the original protocol, the conditioning regimen was required to start within 14 days after completion of chemotherapy. PCIs were classified into two categories: (1) low-intensity PCI, including azacitidine (AZA) and low-dose cytarabine, with or without venetoclax (VEN); and (2) high-intensity PCI, including daunorubicin plus cytarabine, mitoxantrone plus etoposide plus cytarabine, and high-dose cytarabine [10]. Graft-versus-host disease (GVHD) prophylaxis was composed of tacrolimus and methotrexate. Measurable residual disease (MRD) for AML was evaluated by measuring Wilms’ tumor gene-1 (WT1) messenger RNA (mRNA) levels in peripheral blood using a WT1 mRNA assay kit (Otsuka Pharmaceutical Co., Ltd) [11, 12]. WT1 mRNA positivity in peripheral blood was defined as a WT1 mRNA level of ≥ 100 copies/µg RNA. A karyotypic risk classification was based on European LeukemiaNet [13]. Acute and chronic GVHD were defined using consensus criteria [14, 15].

The primary endpoints were overall survival (OS), the cumulative incidence of non-relapse mortality (NRM), and relapse after CBT. The OS was calculated from the date of transplantation to the date of death or last follow-up. Relapse was determined by morphological and clinical evidence of underlying diseases, and NRM was defined as death without relapse. The secondary endpoints were the cumulative incidence of acute and chronic GVHD and hematopoietic recovery. Neutrophil recovery was defined as the first day on which the neutrophil count of 0.5 × 10^9^ /L was reached and sustained over three consecutive measurements.

This was a retrospective observational study. Eligible patients were informed about the purpose and details of the study and were given the opportunity to opt out. The study was approved by the institutional review board of Kanagawa Cancer Center and conducted in accordance with the Helsinki declaration.

### Chimerism analysis

Donor chimerism was assessed using short tandem repeat (STR) analysis, sex-mismatched fluorescence in situ hybridization (FISH), and single-nucleotide polymorphism (SNP) analysis by quantitative PCR. STR analysis was outsourced to SRL Inc., while sex-mismatched FISH and SNP-based qPCR analyses were performed in-house. For STR analysis, full donor chimerism was defined as ≥ 95% donor-derived cells. At our institution, although a formal definition of full donor chimerism had not been established for sex-mismatched FISH or SNP-based qPCR analyses, cases with ≥ 95% donor-derived cells were regarded as full donor chimerism according to institutional practice.

### Statistical analysis

Categorical data were analyzed using the χ^2^ and Fisher’s exact tests, whereas continuous data were analyzed using *t*-tests. The cumulative incidences of relapse, NRM, hematological recovery, and GVHD were analyzed using the gray test. The OS was estimated using a Kaplan–Meier analysis and compared using log-rank tests. Statistical analyses were performed using EZR on R Commander (version 1.55, December 24, 2021) [16].

## Results

### Patient characteristics

This study included 58 patients with a median follow-up duration of 38 months. The median age at CBT was 67 years (range, 52–73), and 29 patients were male. All patients had a performance status of ≤ 2 before CBT, and 33 patients had a Hematopoietic Cell Transplantation–Comorbidity Index of ≤ 2. Poor-risk cytogenetic abnormalities were observed in 15 patients, and 23 patients had non-remission disease status following CBT. WT1 mRNA was observed in 49 patients, with 31 being WT1 mRNA -positive at transplantation (Table 1, Supplemental Table 1). The median time to engraftment was 20 days (range, 14–42), with an engraftment rate of 87.9%. Among the seven patients who did not achieve engraftment by day 42, two underwent a second transplantation and achieved engraftment. In total, 22 patients underwent PCI, the most frequent being VEN + AZA (*n* = 9), and 10 patients underwent post-transplant maintenance therapy.

**Table 1:** Patient characteristics

	FM80TBI (*N*=58)
Median age, years (range)	67 (52-73)
Age ≥ 65, n (%)	47 (81.0)
Sex, n (%)	
Female	29 (50)
Male	29 (50)
AML, n (%)	
De novo	52 (89.7)
Secondary	6 (10.3)
Cytogenetic risk, n (%)	
Favorable	4 (6.9)
Intermediate	39 (67.2)
Adverse	15 (25.9)
Disease status, n (%)	
CR	35 (60.3)
Non CR	23 (39.7)
Median blast in BM at SCT, % (range)	1.0 (0.0-62.6)
Performance status at SCT, n (%)	
0	37 (63.8)
1	17 (29.3)
2	4 (6.9)
HCT-CI, n (%)	
0–2	33 (56.9)
≥ 3	25 (43.1)
WT1 at SCT, n (%)	
Positive	31 (53.4)
Negative	18 (31.0)
No data	9 (15.5)
HLA antibody, n (%)	
Negative	32 (55.2)
Positive DSA -	24 (41.4)
Positive DSA+	0 (0.0)
No data	2 (3.4)
Median number of nucleated cell count in CB, 10⁷/kg (range)	3.1 (1.7–6.7)
Median number of CD34-positive cells in CB, 10⁵/kg (range)	0.8 (0.27–2.6)

Abbreviations: AML, acute myeloid leukemia; BM, bone marrow; CB, cord blood cell; CR, complete remission; DSA, donor-specific antibody; HCT-CI, hematopoietic cell transplantation-specific comorbidity index; HLA, human leukocyte antigen; SCT, stem cell transplantation; WT1, Wilms tumor 1

### Transplant outcomes

**Fig. 1:**
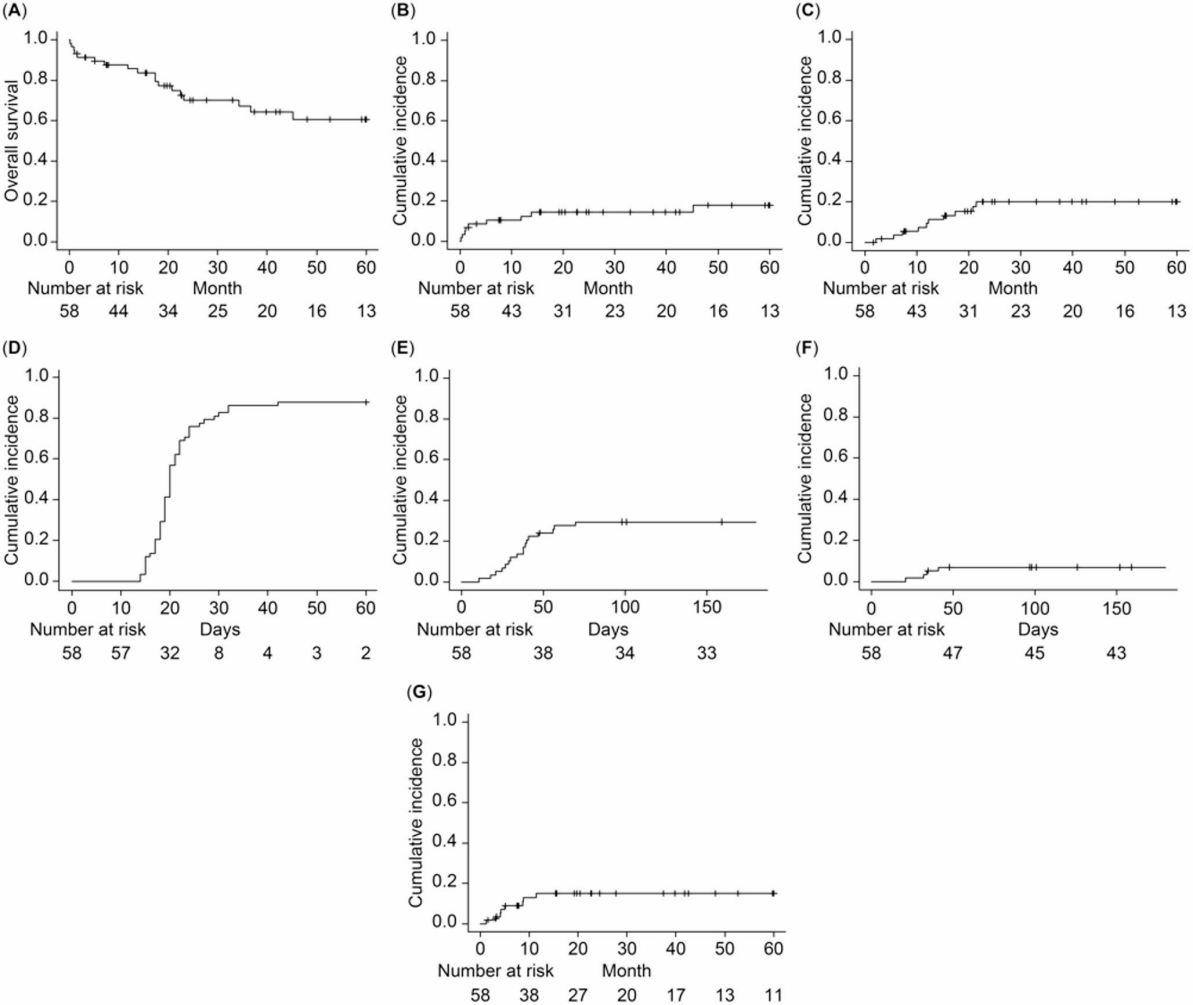
(**A**) Overall survival for the entire cohort. (**B**) Cumulative incidence of NRM for the entire cohort. (**C**) Cumulative incidence of relapses for the entire cohort. (**D**) Engraftment of neutrophils. (**E**) Cumulative incidence of grade II–Ⅳ acute GVHD by day 100 after CBT. (**F**) Cumulative incidence of grade Ⅲ–Ⅳ acute GVHD by day 100 after CBT. (**G**) Cumulative incidence of chronic GVHD by 2 years after CBT. Abbreviations: NRM, non-relapse mortality; GVHD, graft-versus-host disease; CBT, cord blood transplantation

**Fig. 2:**
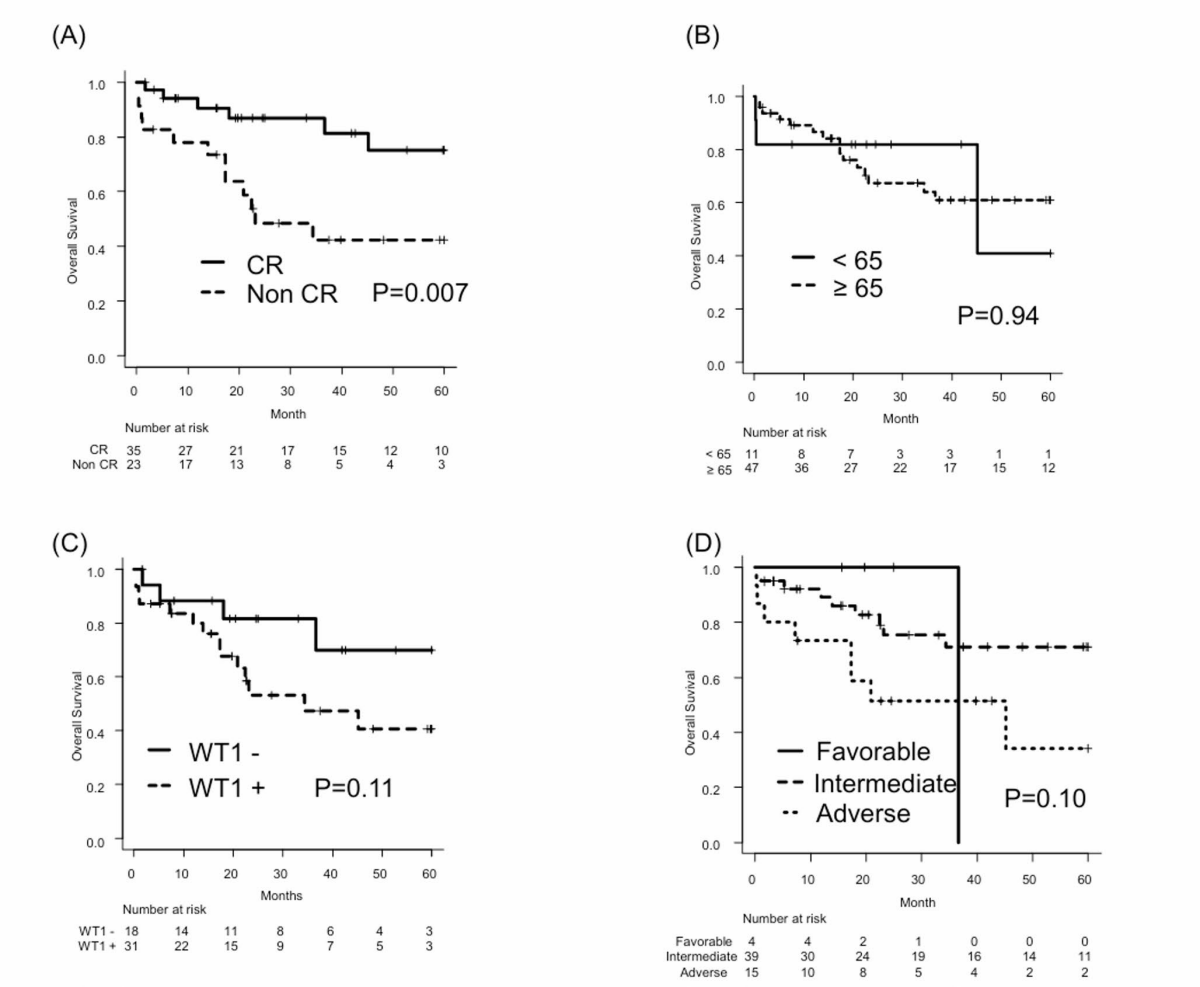
(**A**) Comparison of 3-year OS between patients in CR and those not in CR at the time of CBT. (**B**) Comparison of 3-year OS between patients aged ≥65 years and those aged <65 years at the time of CBT. (**C**) Comparison of 3-year OS between WT1-positive and WT1-negative patients at the time of CBT. (**D**) Comparison of 3-year OS among patients with favorable, intermediate, and adverse cytogenetic risk at diagnosis. Abbreviations: OS, overall survival; CBT, cord blood transplantation; WT1, Wilms tumor 1; CR, complete remission

In the entire cohort, the 3-year OS was 67.1% (95% confidence interval [CI]: 51.5–78.7%) (Fig. 1A). When stratified by disease status following CBT, the 3-year OS was 86.8% (95% CI: 68.3–94.9%) in patients with CR status and 42.4% (95% CI: 20.9–62.5%) in those with non-CR status (*p* = 0.007) (Fig. 2A). No significant differences were observed in terms of age, WT1, or cytogenetic risk (Fig. 2B, C, and D).

The 3-year cumulative incidence of NRM was 14.4% (95% CI: 6.6–25.0%) (Fig. 1B). NRM occurred in nine patients, with causes including septic shock (*n* = 3), pneumonia (*n* = 2), graft failure (*n* = 2), hepatic abscess (*n* = 1), and pneumocystis pneumonia (*n* = 1).

The 3-year cumulative incidence of relapse was 20.0% (95% CI: 10.2–32.3%) (Fig. 1C).

### Hematopoietic recovery and GVHD

The median time to engraftment was 20 days (range, 14–42), and the engraftment rate at day 42 was 87.9% (95% CI: 75.6–94.3%) (Fig. 1D). At day 100 after transplantation, chimerism analysis was not available in eight of the 58 patients because of early post-transplant death, including graft failure or NRM. Among the remaining 50 evaluable patients, 47 had full donor chimerism. The cumulative incidence of acute GVHD by day 100 after CBT was 29.3% (95% CI: 18.2–41.5%) for grade ≥ II and 6.9% (95% CI: 2.2–15.5%) for grade ≥ III (Fig. 1E and F). The 2-year cumulative incidence of chronic GVHD after CBT was 15.0% (95% CI: 6.9–26.0%) (Fig. 1G).

## Discussion

We analyzed the outcomes of initial CBT using FM80TBI as the conditioning regimen in patients with AML and found that transplant outcomes were favorable. The median age of patients in this study was 67 years, reflecting a cohort composed largely of older individuals. Studies have reported that in stem cell transplantation (SCT) for older patients, 3–4 year OS ranges from 30% to 50%, while NRM over the same period is approximately 20% to 40% [17–20]. Although the disease status at the time of SCT, conditioning regimens, and donor sources varied among the studies—making direct comparisons challenging—the transplant outcomes observed in this study were favorable, even in comparison to those reported previously.

Studies on CBT have reported 2- to 3-year OS ranging from 23% to 49.8%, and 2- to 3-year NRM ranging from 25.2% to 53% [21–23]. Although the differences in disease status at the time of CBT, conditioning regimens, and transplant periods make direct comparisons difficult, the transplant outcomes in this study were favorable by comparison. One potential explanation for these favorable outcomes is the lower proportion of patients with non-remission status during CBT in this study, which may have contributed to the improved OS.

Kurita et al. reported a comparison of transplant outcomes among fludarabine-based conditioning regimens used in CBT for myeloid malignancies. The FM140TBI regimen was the most preferred, as it was associated with better OS, leukemia-free survival, a lower relapse rate, and reduced NRM. After adjusting for age, FM140TBI tended to yield better transplant outcomes than FM80TBI [8]. However, Albanyan et al. reported that a melphalan dose of 100 mg/m² was associated with a higher incidence of gastrointestinal toxicity and GVHD compared with a dose of 140 mg/m² [24]. Sakatoku et al. noted that FM80 or FM100 may be less toxic and tissue-destructive than FM140, potentially resulting in fewer infections, bleeding events, and sinusoidal obstructive syndrome [25]. Although FM140TBI may enhance antitumor effects, its use in older patients may be limited because of the increased risk of conditioning-related toxicity and associated NRM. In this study, CBT following FM80TBI demonstrated relatively favorable OS and low NRM, suggesting that FM80TBI may represent a safer conditioning regimen for older patients. Future studies should aim to identify suitable subsets of older patients for FM140TBI.

In this study, the engraftment rate and NRM were comparable to those reported in previous studies. Kurita et al. noted that, fludarabine/melphalan regimen was associated with a higher engraftment rate compared with the fludarabine/busulfan regimen, which may have contributed to a reduction in infections and a decrease in NRM [8]. Melphalan, an alkylating agent, exerts both immunosuppressive and myeloablative effects [26], making it effective for facilitating engraftment and achieving tumor control. Given the issue of engraftment failure in CBT, the fludarabine/melphalan regimen is considered a favorable option.

Duval et al. reported that the 3-year OS after SCT for relapsed or non-CR AML was 19% [27]. In this study, the 3-year OS for patients with non-CR AML was lower than that of those with CR AML; however, it was more favorable than previously reported. Ogawa et al. reported that among patients with non-CR AML, those with 0% peripheral blood blasts and < 20% bone marrow blasts at the time of SCT had a relatively favorable prognosis [28]. At our center, PCI is administered before the initiation of conditioning in patients with non-CR AML, with the aim of reducing tumor burden [10]. In this study, PCI was administered in 22 cases, which may have contributed to the more favorable outcomes observed in patients with non-CR AML compared with previous reports. These findings underscore the potential role of PCI in reducing disease burden before conditioning, highlighting the importance of pre-transplant optimization strategies, especially in patients with active disease [10].

This study has some limitations. First, as a retrospective study, selection bias cannot be excluded. Second, owing to the small number of events, a multivariate analysis was not performed. Further clinical trials are needed to establish the efficacy and safety of FM80TBI.

In conclusion, this single-center study demonstrated favorable outcomes of CBT using FM80TBI in older patients with AML or those with significant comorbidities.

## Electronic Supplementary Material

Below is the link to the electronic supplementary material.

Supplementary Material 1

## Data Availability

For original data, please contact the corresponding author.

## Declarations

### Permission to reproduce material from other sources

N/A.
