# Supplementary material for: Outcome of initial cord blood transplantation with FM80TBI as conditioning regimen for acute myeloid leukemia
Source: Ann Hematol. 2026 Feb 16;105(4):118. doi: 10.1007/s00277-026-06888-3 (PMC12909624; doi:10.1007/s00277-026-06888-3)
Supplement: Supplementary file 1 — Supplementary Material 1 [file 277_2026_6888_MOESM1_ESM.docx]

Supplemental Table1. Details of chromosomal abnormalities

| Cytogenetic risk, n (%) | N=58 |
| --- | --- |
| Favorable | 4 |
| inv(16)(p13.1q22) | 1 |
| t(8;21)(p22;q22.1) | 3 |
| Intermediate | 39 |
| Normal karyotype | 29 |
| Not Normal karyotype | 10 |
| Poor | 15 |
| -7 | 2 |
| t(v;11)(v;q23) | 1 |
| inv(3)(q21.3q26.2) | 1 |
| Complex karyotype | 11 |

Abbreviations: inv, inversion
